# Supplementary material for: Detection of de novo copy number deletions from targeted sequencing of trios
Source: Bioinformatics. 2018 Aug 2;35(4):571–8. doi: 10.1093/bioinformatics/bty677 (PMC6378941; doi:10.1093/bioinformatics/bty677)
Supplement: Supplementary Material [file bty677_supplementary_material.pdf]

SUPPLEMENTARY MATERIAL

Detection of de novo copy number deletions from targeted sequencing of trios

Jack M. Fu[1], **Elizabeth J. Leslie**[2], **Alan F. Scott**[3], **Jeffrey C. Murray**[4], **Mary L. Marazita**[5], **Terri H. Beaty**[6], **Robert B. Scharpf**[7], **Ingo Ruczinski**[1*]

[1] Department of Biostatistics, Johns Hopkins University, Baltimore MD.

[2] Department of Human Genetics, Emory University, Atlanta GA.

[3] Institute of Genetic Medicine, Johns Hopkins School of Medicine, Baltimore MD.

[4] Department of Pediatrics, Carver College of Medicine, University of Iowa, Iowa City, IA.

[5] School of Dental Medicine, University of Pittsburgh, Pittsburgh PA.

[6] Department of Epidemiology, Johns Hopkins University, Baltimore MD.

[7] Department of Oncology, Johns Hopkins University, Baltimore MD.

[*]To whom correspondence should be addressed: Department of Biostatistics, Johns Hopkins Bloomberg School of Public Health, 615 North Wolfe Street, Baltimore MD, 21205, USA. Email ingo@jhu.edu.

size (bp) ⟶	250	500	1,000	2,000	4,000
MDTS	182	465	825	948	986
MDTS:p	304	444	627	824	903
CANOES:b	360	729	958	995	995
CANOES	498	640	783	905	965

Supplementary Table 1: Number of true positive identifications of *de novo* deletions (sensitivity) among 1,000 iterations in the simulation study. MDTS and CANOES refer to the respective algorithms as implemented, MDTS:p refers to MDTS based on the "probe-based" bins, CANOES:b refers to CANOES based on the non-uniform read depth based bins.

size (bp) ⟶	250	500	1,000	2,000	4,000
MDTS	0	1	1	1	0
MDTS:p	3	3	7	3	2
CANOES:b	87	156	144	63	16
CANOES	78	47	32	19	2

Supplementary Table 2: Number of false positive identifications among the inherited deletions, among 1,000 iterations in the simulation study, for four different algorithms.

	N	Median	Minimum	Maximum
MDTS	2	506	374	637
MDTS:p	1,944	241	121	8,243
CANOES:b	114	2,485	206	19,709
CANOES	2,967	361	121	24,474

Supplementary Table 3: The total number N of incorrectly called *de novo* deletions among 1,000 iterations in the simulation study, and median, minimum, and maximum sizes (in nucleotide bases) among those false positives, for four different algorithms.

size (bp) ⟶	250	500	1,000	2,000	4,000
MDTS	0.989	0.996	0.998	0.998	0.998
MDTS:p	0.135	0.186	0.244	0.298	0.317
CANOES:b	0.759	0.865	0.894	0.897	0.897
CANOES	0.144	0.177	0.209	0.234	0.245

Supplementary Table 4: Estimated positive predictive values for the four algorithms.

	start	end	size	n_{hom}	n_{het}
chromosome 1	210,078,417	210,085,527	7,111	297	507
chromosome 8	129,762,791	129,766,015	3,225	296	450

Supplementary Table 5: Two regions, close to the inferred *de novo* deletions, were highly polymorphic for CNVs in the oral cleft TS data. The data indicate approximate genomic coordinates and the size (in base pairs) of the CNPs, as well as the number of probands with an inherited homozygous (n_{hom}) or heterozygous (n_{het}) deletion.

	MDTS		15-core MDTS		CANOES		TrioCNV	
		:p		:p	:b		:b	
Binning	2		2		2		2	
Counting	25	25	3	4	600	1,180	*25	*25
Inference	2	12	1	1	*70	*130	5	9
Total	29	37	6	5	672	1,310	32	34

Supplementary Table 6: Runtimes (CPU hours, rounded) of MDTS, CANOES, and TrioCNV, and respective modified versions thereof, on the full dataset of 1,018 oral cleft trios, plus runtime for MDTS using an embarrassingly parallel multi-threaded version using 15 cores. Binning refers to the read depth based delineation of MDTS bins using a randomly selected subset of samples, as described in the Methods section. Counting refers to the calculation of read depths for the bins used in the respective algorithms. The asterisk (*) indicates that modifications were made to the publicly available code, described in detail in the Methods section.

	MDTS		15-core MDTS		CANOES		TrioCNV	
		:p		:p	:b		:b	
Binning	15		15		15		15	
Counting	11	14	160	200	1	1	*11	*11
Inference	7	15	105	210	*6	*14	2	3
Maximum	15	15	160	210	15	14	15	11

Supplementary Table 7: Memory requirements (GB) of MDTS, CANOES, and TrioCNV, and respective modified versions thereof, on the full dataset of 1,018 oral cleft trios, plus memory requirements for MDTS using an embarrassingly parallel multi-threaded version using 15 cores. Binning refers to the read depth based delineation of MDTS bins using a randomly selected subset of samples, as described in the Methods section. Counting refers to the calculation of read depths for the bins used in the respective algorithms. The asterisk (*) indicates that modifications were made to the publicly available code, described in detail in the Methods section.

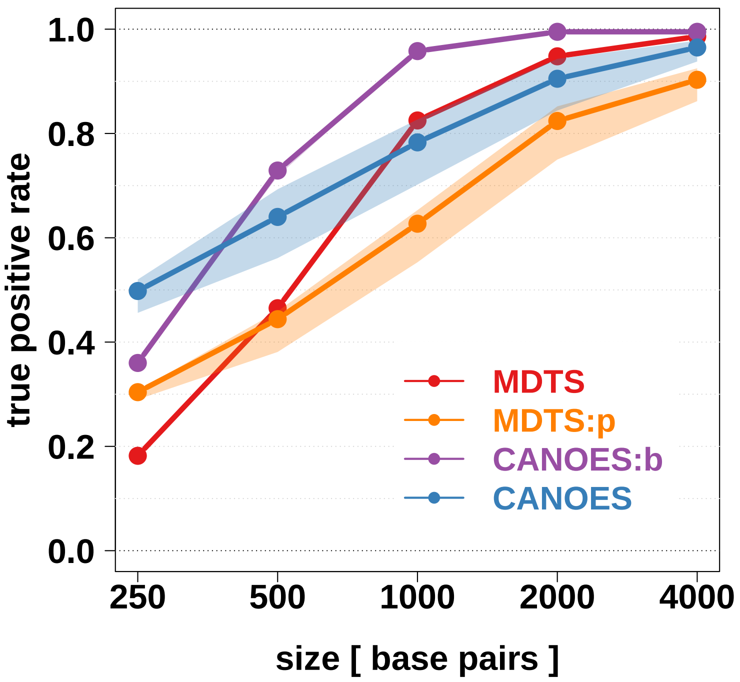

Supplementary Figure 1: True positive rate (sensitivity, y-axis) among 1,000 iterations for simulated *de novo* deletions of various sizes (x-axis) using different definitions of ”overlap” to define true positives, for four different algorithms. The lines show true positive rates using the 25% threshold described in the Methods and shown in Figure 2. The top of the bands result from using a >0% threshold (e.g., any overlap), the bottom of the bands result from a 50% threshold (e.g. at least half of the deletion was identified). The true positive rate estimates essentially do not depend on the threshold when MDTS bins are used.

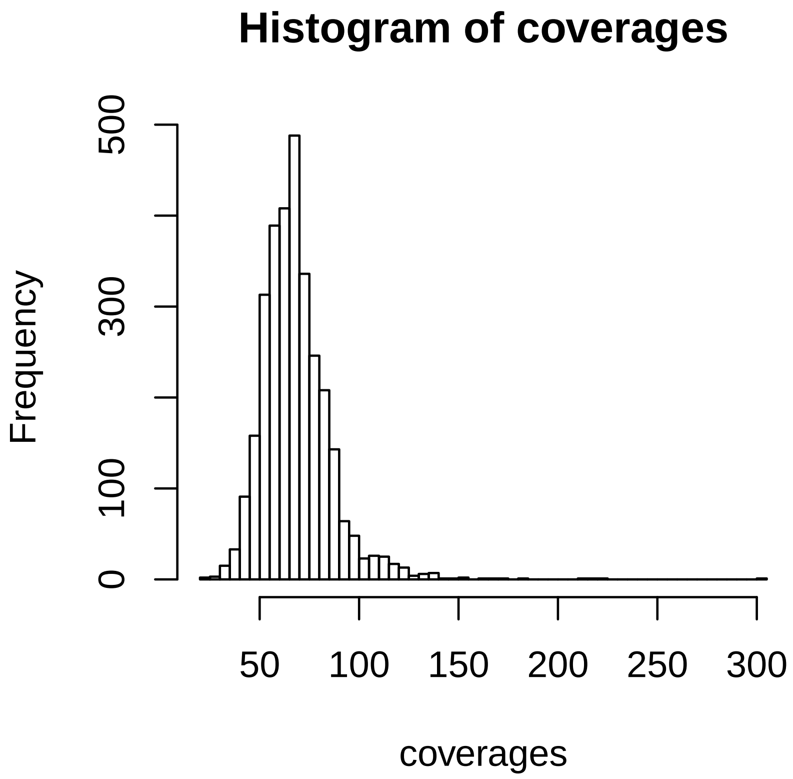

Supplementary Figure 2: Average coverage among the 3,054 samples analyzed in the oral cleft case study, across the 6.3Mb region identified by the MDTS binning procedure.

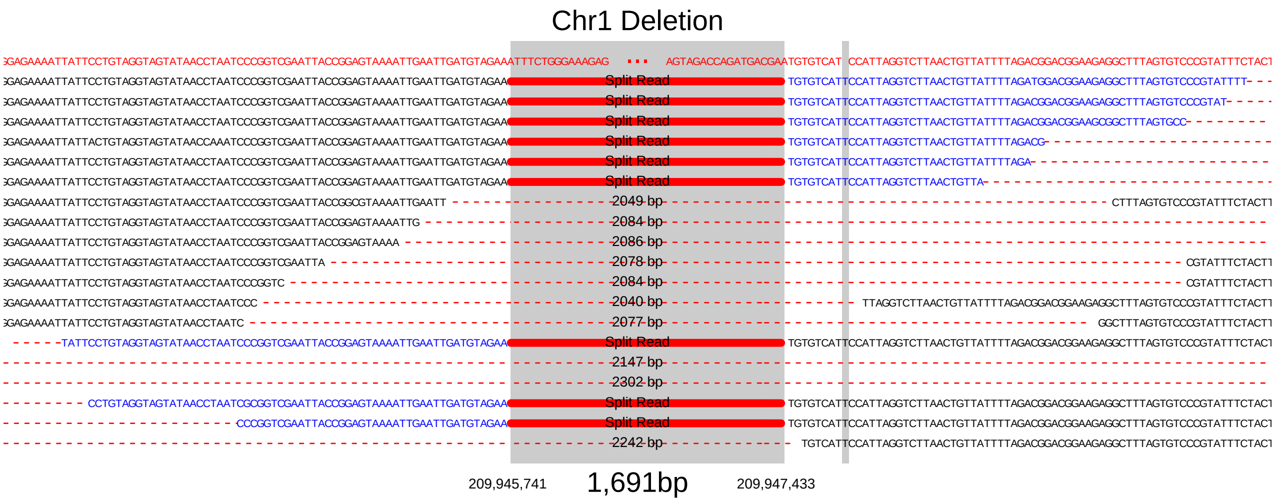

Supplementary Figure 3: Evidence for the *de novo* deletion in the proband of family DS10826 from WGS data with 151bp paired-end reads. The breakpoints are at 209,945,742 and 209,947,432 on chrosome 1, indicating a 1,691 base pairs segment. Among 19 improper paired-end reads were 9 where one read of the pair was split across a breakpoint (the primary piece of these BWA-aligned reads are shown in black, the clipped reads were manually aligned and are shown in blue). The remaining 10 read pairs do not explicitly reveal the breakpoint, but the fragment sizes between the ends are all well above 2kb, further supporting the existence of the deletion. The reference genome (reverse complemented) is shown in red at the top, and reveals an insertion of a second "T" after 209,947,950 (grey vertical bar). This insertion is also *de novo*, as it was not observed in either parent.

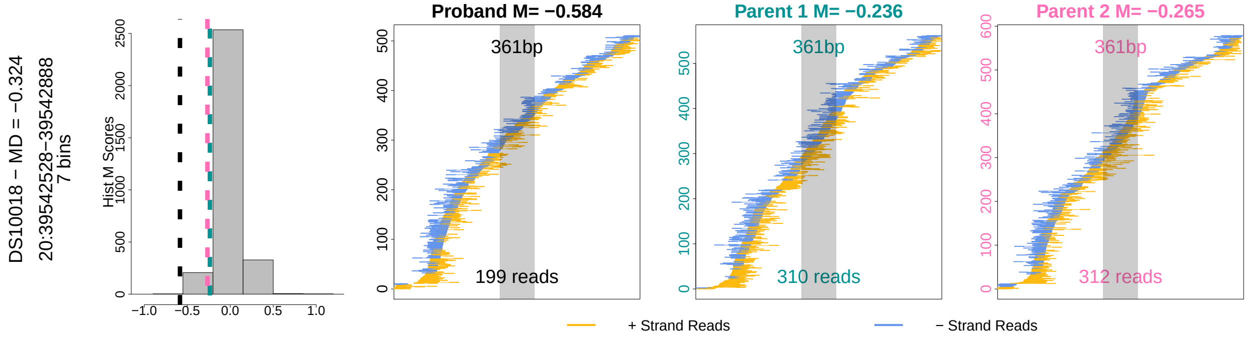

Supplementary Figure 4: 2,702 of the 2,970 trios with CANOES inferred proband *de novo* deletion did not have Minimum Distances consistent with such events. In this example the Minimum Distance was -0.32. The proband does not have discordant read pairs flanking the identified 361bp region.

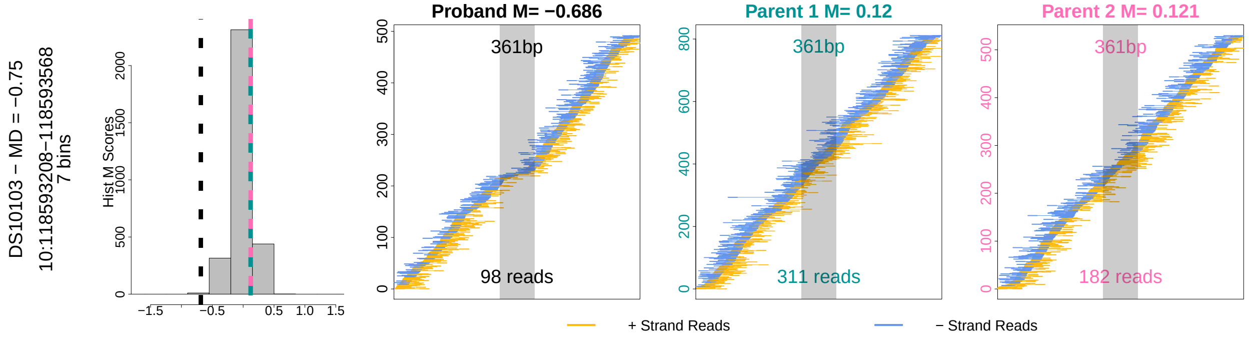

Supplementary Figure 5: 267 trios with CANOES inferred proband *de novo* deletion did have Minimum Distances consistent with such events. In this example the Minimum Distance was -0.75. However, in none of these trios discordant read pairs flanking the identified regions were present.

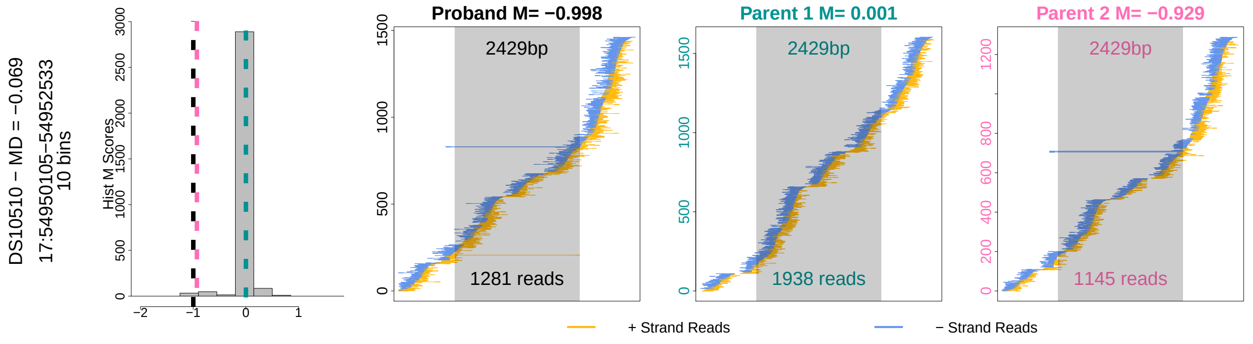

Supplementary Figure 6: A Mendelian event incorrectly called *de novo* by CANOES:b. The M scores (-1.00, 0.00, and -0.93 for the proband and the parents, respectively) and the family Minimum Distance of -0.07 indicate an inherited heterozygous deletion from parent 2. This is further corroborated by the read "Z signatures" in the proband and parent 2.

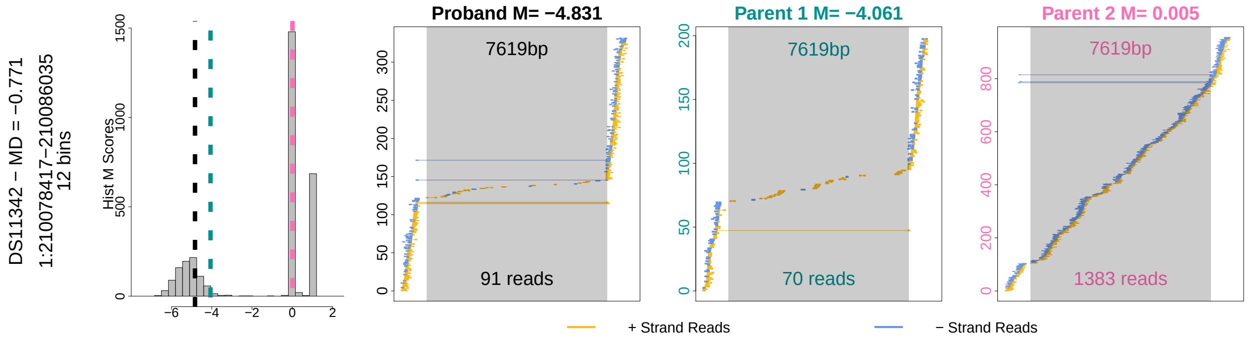

Supplementary Figure 7: A Mendelian event incorrectly called *de novo* by CANOES:b. The M scores (-4.83, -4.06, and 0.01 for the proband and the parents, respectively) and the family Minimum Distance of -0.77 indicate an inherited homozygous deletion in the proband, from one homozygous parent (1) and one heterozygous parent (2). This is further corroborated by the read "Z signatures" in the individuals.

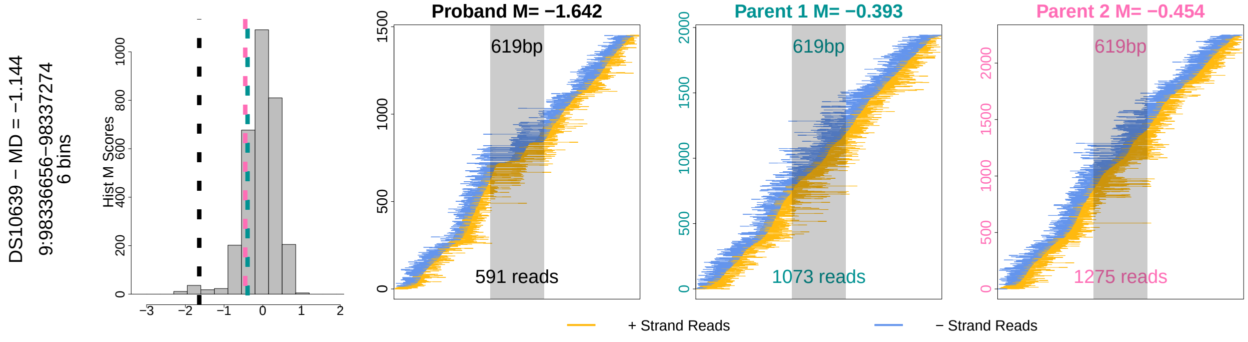

Supplementary Figure 8: A technical (likely read mapping) artifact, resulting in a *de novo* call by CANOES:b. This pattern is observed in many samples, and reflected in the variability of the M score distribution. Although the Minimum Distance is -1.14, this region is discarded by MDTS due to the spread of the M scores.

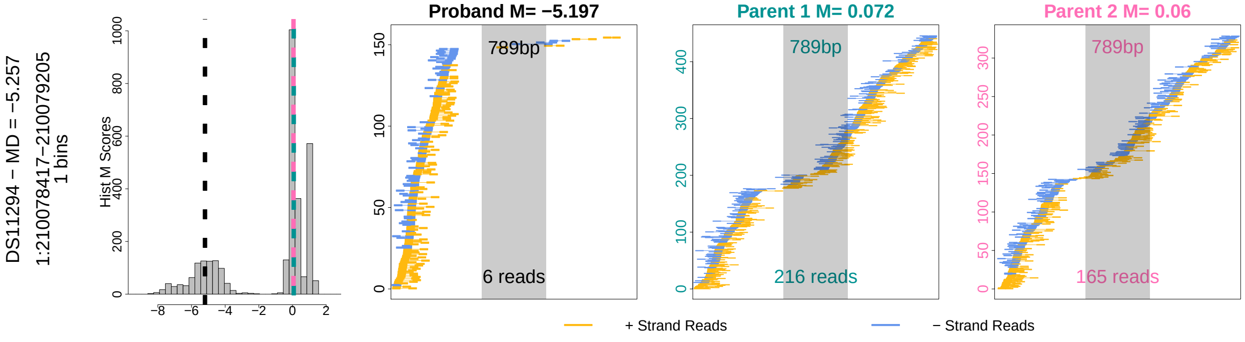

Supplementary Figure 9: A Mendelian event incorrectly called *de novo* by TrioCNV:b. The data clearly indicate a homozygous deletion in the proband, resulting through inheritance of one heterozygous deletion from each parent. This region is actually a piece of the larger CNP on chromosome 1 identified by MDTS.

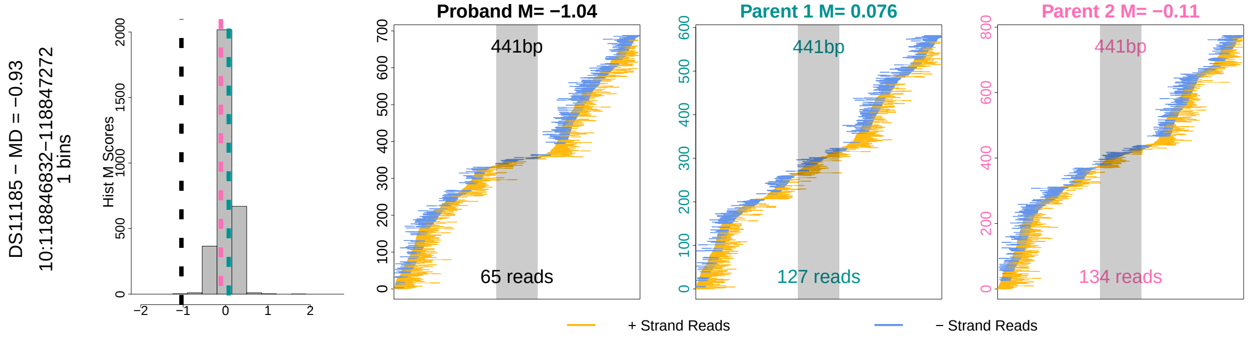

Supplementary Figure 10: The M scores (-1.04, 0.08, and -0.11 for the proband and parents respectively) and the resulting Minimum Distance of -0.93 are consistent with a *de novo* deletion, very few reads are observed in this reqion, resulting in one bin only and highly variable statistics. In addition, no discordant read-pairs span this 441bp region.
